# Comparative Metabolite Fingerprinting of the Rumen System during Colonisation of Three Forage Grass (*Lolium perenne* L.) Varieties

**DOI:** 10.1371/journal.pone.0082801

**Published:** 2013-11-27

**Authors:** Alison H. Kingston-Smith, Teri E. Davies, Pauline Rees Stevens, Luis A. J. Mur

**Affiliations:** Institute of Biological, Environmental and Rural Sciences, Aberystwyth University, Aberystwyth, United Kingdom; University of Illinois, United States of America

## Abstract

The rumen microbiota enable ruminants to degrade complex ligno-cellulosic compounds to produce high quality protein for human consumption. However, enteric fermentation by domestic ruminants generates negative by-products: greenhouse gases (methane) and environmental nitrogen pollution. The current lack of cultured isolates representative of the totality of rumen microbial species creates an information gap about the *in vivo* function of the rumen microbiota and limits our ability to apply predictive biology for improvement of feed for ruminants. In this work we took a whole ecosystem approach to understanding how the metabolism of the microbial population responds to introduction of its substrate. Fourier Transform Infra Red (FTIR) spectroscopy-based metabolite fingerprinting was used to discriminate differences in the plant-microbial interactome of the rumen when using three forage grass varieties (*Lolium perenne* L. cv AberDart, AberMagic and Premium) as substrates for microbial colonisation and fermentation. Specific examination of spectral regions associated with fatty acids, amides, sugars and alkanes indicated that although the three forages were apparently similar by traditional nutritional analysis, patterns of metabolite flux within the plant-microbial interactome were distinct and plant genotype dependent. Thus, the utilisation pattern of forage nutrients by the rumen microbiota can be influenced by subtleties determined by forage genotypes. These data suggest that our interactomic approach represents an important means to improve forages and ultimately the livestock environment.

## Introduction

Rumen fermentation is complex, involving a diverse population of bacteria, fungi, protozoa and archaea, the proportions of which can be affected by feed type and quality [1,2]. A key target for sustainable animal production is to develop a more complete understanding of the functioning of the rumen as an ecosystem which will allow informed manipulations of the rumen, at either microbiota or feed level, by which to maximise productivity whilst minimising environmental impact [3]. Evaluation of the composition of the ruminal microbial population has revealed that estimates of numbers of taxa of ruminal microbiota based on nucleic acid-based methodologies vastly exceed those (approximately 15%) that have been cultured and characterised [4–7]. The richness of population diversity can provide resilience of the rumen system to perturbations imposed by managed intervention such as change in dietary forage, feed additive or probiotic supplementation [8–12]. There is also the possibility that *in vitro* and *in vivo* activities are dissimilar due to physical and compositional community structure. For instance Czerkawski and Cheng described three components to the rumen microbiota [13]: those associated with the rumen wall, those firmly associated with the feed particles (degradative compartment) and those reversibly associated with food particles (the shuttle compartment). Together, these factors present a considerable challenge in terms of understanding microbial community nutritional requirements and how the microbial community interacts to digest its substrate. 

In the grazing situation, fresh forage offers the rumen microbiota a chemically rich substrate with components of varying solubility, digestibility and antimicrobial activity which will determine primary colonisation. As well as the chemical composition, fresh forage is metabolically active and will induce cellular stress and defence responses to the temperature (39 °C) and anaerobic conditions of the rumen, including induction of proteolysis [14–19], a switch from respiratory to fermentative ATP generation [20], and plant hormone based changes in defence gene expression [21]. Spatio-temporal variation in such metabolic activities in the different biological entities represents a plant-microbe interactome within the rumen ecosystem which is far too complex to approach with standard food web modelling techniques. 

 Chemical fingerprinting techniques are powerful tools in systems-based evaluations. Fingerprinting has previously been successfully applied to quality control for example to identify instances of olive oil adulturation [22]. Recently the potential of this technology for deconvolution of complex, interacting systems has been explored in the context of “dual metabolomics” to examine the metabolism of both host and pathogen during plant pathogenesis [23]. The “dual metabolomics” approach exploited a co-cultivation approach to track metabolite changes occurring within each of the partners of the interactome arising as a result of reactions of *Arabidopsis* cells with virulent and avirulent strains of *Pseudomonas syringae* [23]. In this study, Fourier Transform Infra-Red (FTIR) spectroscopy was employed as a high-throughput metabolite fingerprinting approach. FTIR fingerprinting is non-discriminatory in primary detection parameters, so can be used to infer interactions within the interactome that might be overlooked by targeted analysis. In this current study, we have investigated the principles of dual metabolomics as applied to the plant-microbial interactome of the rumen during colonisation and fermentation of different but related forage grass varieties. FTIR fingerprint spectra were interrogated using multivariate analysis which identified those spectral regions that were associated with major variabilities between samples. Crucially, changes could be linked to plant genotype-specific differences in nutrient flux to the microbial community within the interactome. Thus, our interactome approach should be exploited to inform plant breeding strategies and offers a platform on to which to build a systems biology approach to understanding rumen function. 

## Materials and Methods

### Plant material

*Lolium perenne* L. varieties Premium, AberDart and AberMagic were each grown from seed in replicate trays (21 x 34 x 5 cm) containing Levingtons Multipurpose Compost (Levingtons, UK) for 6 weeks in a growth cabinet (22 °C/ 18 °C day/night temperature, 8 h photoperiod with a light level of 300 µmol m^-2^s^-1^) which were watered regularly. Grass from independent replicate trays was harvested by cutting with scissors approx 5 cm above the soil surface, and subsequently cut further into 0.5-1 cm lengths. 

### Collection and preparation of microbial inoculum

Experiments were conducted under the authority of licenses under the U.K. Animal Scientific Procedures Act, 1986. Rumen fluid was collected from each of four non-lactating Holstein-Friesian dairy cows fed ad-lib on 54 % dry matter grass silage (perennial ryegrass *Lolium perenne* cv AberStar; composition 1.64 % N, 4.89 % water soluble carbohydrate (WSC), 55.63 % neutral detergent fibre (NDF), 30.76 % acid detergent fibre (ADF) and 5.37 % acid digestible lignin (ADL), and which had been previously prepared with rumen cannulae (Bar Diamond, Parma, ID). The combined sample was filtered through two layers of muslin under a CO_2_ stream and the filtrate used to prepare a 10 % rumen fluid inoculum in anaerobic buffer [24], which was pre-warmed to 39 °C.

### Experimental design

For each grass genotype 0.5 g of cut grass was placed into each of 24 Hungate tubes. Tubes were filled with CO_2_ prior to inoculation with 5 ml of the 10 % rumen fluid inoculum (under a stream of CO_2_). Tubes were back-filled with CO_2_ and sealed with butyl rubber caps. Tubes were placed in a water bath at 39 °C in the dark. At each sampling point (0, 2, 4, 6, 12 and 24 h post inoculation) four replicate tubes were removed and the contents mixed by inversion. Two aliquots of 1 ml were removed from each incubation for analysis of volatile fatty acids (VFA) and ammonia. The remainder of the contents were separated into three fractions (Figure S1): the cell free medium (referred to as the footprint), the bacteria in the planktonic phase (referred to as the pellet) and the plant residue (referred to as the residue). An aliquot of 2 ml of incubation fluid was filtered through two layers of muslin (to collect plant debris but permit passage of bacteria) into a microfuge tube, which was centrifuged at room temperature for 10 min at 10,000 x g. The upper 0.5 ml was removed and placed in a clean microfuge tube and stored at -80 °C until use for analysis of the footprint. The remaining supernatant was removed and discarded before washing the pellet in 0.5 ml deionised water. The water was removed and discarded from the pellet sample, which was frozen in liquid nitrogen and stored at -80 °C until use. The remaining 3 ml of the incubation (incubation fluid plus plant material) was vacuum filtered through Whatman no. 1 filter paper. Residues were retained on the filter paper and washed with approximately 50 ml deionised water before being placed in 1.5 ml microfuge tubes, frozen in liquid nitrogen and stored at -80 °C until use (Figure S1).

### Chemical analysis

Forages used in the experimentation were subject to compositional analyses of N, WSC, fibre (NDF, ADF) and lignin (ADL) as described previously [25]. Samples for VFA analysis were acidified by the addition of 4 % orthophosphoric acid (final concentration) and analysed by gas chromatography with 4 mM ethylbutyric acid as an internal standard as described previously [26]. Samples for ammonia-N analysis were acidified by addition of conc HCl (5 % v/v final concentration) and analysed by segmented flow analyser as described previously [27]. Independent replicates of footprint samples plus aliquots of incubation buffer and rumen fluid inoculum were analysed in transmission mode by FTIR (Equinox 55 HTS-XT FTIR Spectrophotometer, Bruker UK Ltd, Coventry, UK). For footprint analysis, an aliquot of 10 µl of sample was spotted on to a well of a 96 well silicone plate and dried at 40 °C before scanning. Residue samples were freeze dried and ground to a fine powder in a ball mill (MM 30, Retsch Gmbh, Haan, Germany) at speed 30 for 2 min with the inclusion of 2 tungsten beads previously washed in acetone. A well-mixed sub sample was loaded on to the sample window of the golden gate attachment of the FTIR and scanned. The bacterial pellet samples were resuspended in 200 µl molecular grade water. Samples were homogenised in a ball mill as described above for residue samples. An aliquot of 10 µl of sample was spotted on to a well of a 96 well silicone plate and dried at 40 °C before scanning. All samples were analyzed singly with 10 % of the samples re-analyzed for quality control purposes. The FTIR spectra obtained were converted to xy data for further analysis.

### Data analysis

Differences in forage composition were determined by ANOVA run in either Genstat (15th edition, VSN International Ltd; Hemel Hemstead, UK, [28]) or Minitab (14th Edition). Significant differences between means were detected by Duncan’s multiple range comparison in Genstat. XY data matrices generated from FTIR analysis were exported in ASCII format. The spectra were mined as described in [23]. Data were examined using principal components analysis (PCA) using MATLAB version 6.5 (The MathWorks Inc., Natwick, MA, US) or Pychem software [29].

## Results

Standard compositional analysis of the three *L. perenne* varieties (Table 1) revealed no significant differences between them in terms of total nitrogen (% N), soluble carbohydrate (WSC), neutral detergent fibre (NDF) or acid digestible lignin (ADL). Significant differences in acid detergent fibre (ADF) were detected between the genotypes with ADF being significantly greater in Premium than in AberDart or AberMagic (Table 1). 

**Table 1:** Compositional analysis of *Lolium perenne* cultivars Premium, AberDart and AberMagic.

Genotype	% N	% WSC	% NDF	% ADF	% ADL
Premium	3.53	4.30	38.75	24.48**^*b*^**	5.00
AberMagic	4.04	3.63	37.58	22.64**^*a*^**	7.2
AberDart	4.09	3.94	36.70	23.32**^*a*^**	8.49
Significance	NS	NS	NS	*	NS
Lsd	0.674	1.932	1.921	0.938	5.488

The results are the mean values of samples taken from n = 4 independent trays of grass analysed by one way ANOVA (9 df). Lsd, least significant difference; NS, non significant; * P <0.01. Where significant differences between means were detected, values within a column with different superscripts were significantly different, P <0.05.

The fermentation end products, ammonia and VFA, were measured at intervals after the addition of rumen fluid inoculum to the chopped ryegrasses. The VFA acetate, propionate, butyrate, valerate, caproate and heptanoate were detected in these fermentations (Table 2). There was no detectable effect of genotype on the relative abundance of the individual VFA but an effect of time was detected in all except for valerate (Table 2). Accumulation of VFA was observed in all incubations, with significantly more total VFA production observed in incubations with Premium and AberMagic than with AberDart (Table 2). Accumulation of total and individual VFA was significantly different between early and late stages of fermentation although the exact timing varied by individual VFA; for example acetate was only significantly different to previous timepoints at 24 h whereas propionate was significantly lower at 0 and 2 h than at all other timepoints (Table 2). Accumulation of ammonia was observed during fermentation of all three grass genotypes. This was significantly affected by time and genotype and there was an interaction between time and genotype (Table 3). While ammonia content of fermentation tubes was similar until 6 h, the amounts at 12 h exceeded those at 6 h and amounts at 24 h exceeded those at 12 h. Furthermore, at 24 h the ammonia in fermentations of AberMagic significantly exceeded (by 25-30 %) that detected in the fermentations of either Premium or AberDart (Table 3). 

**Table 2:** Accumulation of VFA during fermentation of three ryegrass genotypes.

Genotype	Incubation time (h)	Total VFA (mM)	Molar ratio (%)					
			Acetate	Propionate	Butyrate	Valerate	Caproate	Heptanoate
**Premium**	0	5.52**^*a*^**	60.75**^*c*^**	16.99**^*a*^**	16.26**^*e*^**	4.47	1.54**^*a*^**	0**^*a*^**
	2	6.69^abc^	57.56^bc^	17.66^ab^	13.84^bcde^	8.04	2.88^abc^	0**^*a*^**
	4	8.46^bcde^	60.40**^*c*^**	20.40^efg^	13.06^bc^	3.69	2.45^ab^	0**^*a*^**
	6	10.44^efg^	59.91**^*c*^**	21.16^efg^	12.65^ab^	3.35	2.92^abc^	0**^*a*^**
	12	16.79**^*h*^**	57.30^bc^	22.23**^*g*^**	13.53^bcd^	3.79	3.15^abc^	0**^*a*^**
	24	26.76**^*j*^**	50.53**^*a*^**	21.21^efg^	13.22^bcd^	5.21	5.83**^*f*^**	4.00^ab^
**AberMagic**	0	6.38^abc^	59.67**^*c*^**	16.89**^*a*^**	15.74^de^	4.38	3.33^bc^	0**^*a*^**
	2	7.72^abcd^	59.20**^*c*^**	18.31^abcd^	14.39^bcde^	4.09	4.01^bcde^	0**^*a*^**
	4	9.63^def^	60.12**^*c*^**	20.02^def^	12.76^ab^	3.72	3.38^bcd^	0**^*a*^**
	6	12.43**^*g*^**	59.18**^*c*^**	21.55^fg^	12.32^ab^	3.48	2.96^abc^	0.50**^*a*^**
	12	17.34**^*h*^**	58.52**^*c*^**	21.01^efg^	13.42^bcd^	3.60	2.75^abc^	0.70**^*a*^**
	24	27.89**^*j*^**	52.17^ab^	20.79^efg^	13.15^bcd^	5.49	5.00^def^	3.40^ab^
**AberDart**	0	6.15^ab^	58.81**^*c*^**	16.75**^*a*^**	15.70^cde^	4.35	4.40^cdef^	0**^*a*^**
	2	6.93^abc^	59.36**^*c*^**	17.92^abc^	14.49^bcde^	4.25	3.97^bcde^	0**^*a*^**
	4	8.64^cde^	59.54**^*c*^**	19.72^cdef^	13.35^bcd^	3.85	3.53^bcd^	0**^*a*^**
	6	11.08^fg^	59.83**^*c*^**	20.69^efg^	12.86^ab^	3.56	3.05^abc^	0**^*a*^**
	12	15.94**^*h*^**	53.63^ab^	19.38^bcde^	10.41**^*a*^**	7.32	2.62^ab^	6.65**^*b*^**
	24	24.17**^*i*^**	51.35^ab^	20.98^edg^	13.08^bc^	5.35	5.51^ef^	3.73^ab^
**Significance** (Lsd)								
Genotype		* (0.885)	NS (1.824)	NS (0.745)	NS (0.914)	NS (1.490)	NS (0.582)	NS (1.868)
Time		*** (1.251)	*** (2.579)	*** (1.053)	*** (1.293)	NS (2.107)	*** (0.828)	* (2.642)
Genotype x Time		NS (2.167)	NS (4.467)	NS (1.824)	NS (2.239)	NS (3.649)	NS (1.434)	NS (4.576)

Means of n = 4 samples taken from independent trays of grass are shown. Means within data columns were compared by two way ANOVA (52 df). Lsd, least significant difference; NS, non significant; * P < 0.05, ** P <0.01, *** P <0.001. Where significant differences between means were detected , values with different superscripts within a column were significantly different, P <0.05.

**Table 3:** Accumulation of ammonia (mg/l NH_3_-N) during fermentation of *Lolium perenne* varieties Premium, Aberdart and Abermagic.

	Incubation time (h)					
Genotype	0	2	4	6	12	24
Premium	17.8**^*a*^**	25.9^ab^	36.2^abc^	43.1^abc^	73.4^de^	114.3^fg^
AberMagic	22.2**^*a*^**	27.0^abc^	38.4^abc^	55.0^cd^	93.8^ef^	156.9**^*h*^**
AberDart	21.6**^*a*^**	26.0^ab^	32.3^abc^	52.4^bcd^	77.6^de^	124.3**^*g*^**
**Significance** (Lsd)						
Genotype	*** (6.27)					
Time	*** (8.87)					
Genotype x time	* (15.36)					

Means of n = 4 samples taken from independent trays of grass were compared by two way ANOVA (54 df). Lsd, least significant difference; NS, non significant; * P < 0.05, ** P <0.01, *** P <0.001. Values with different superscripts were significantly different, P <0.05.

**Figure 1:**
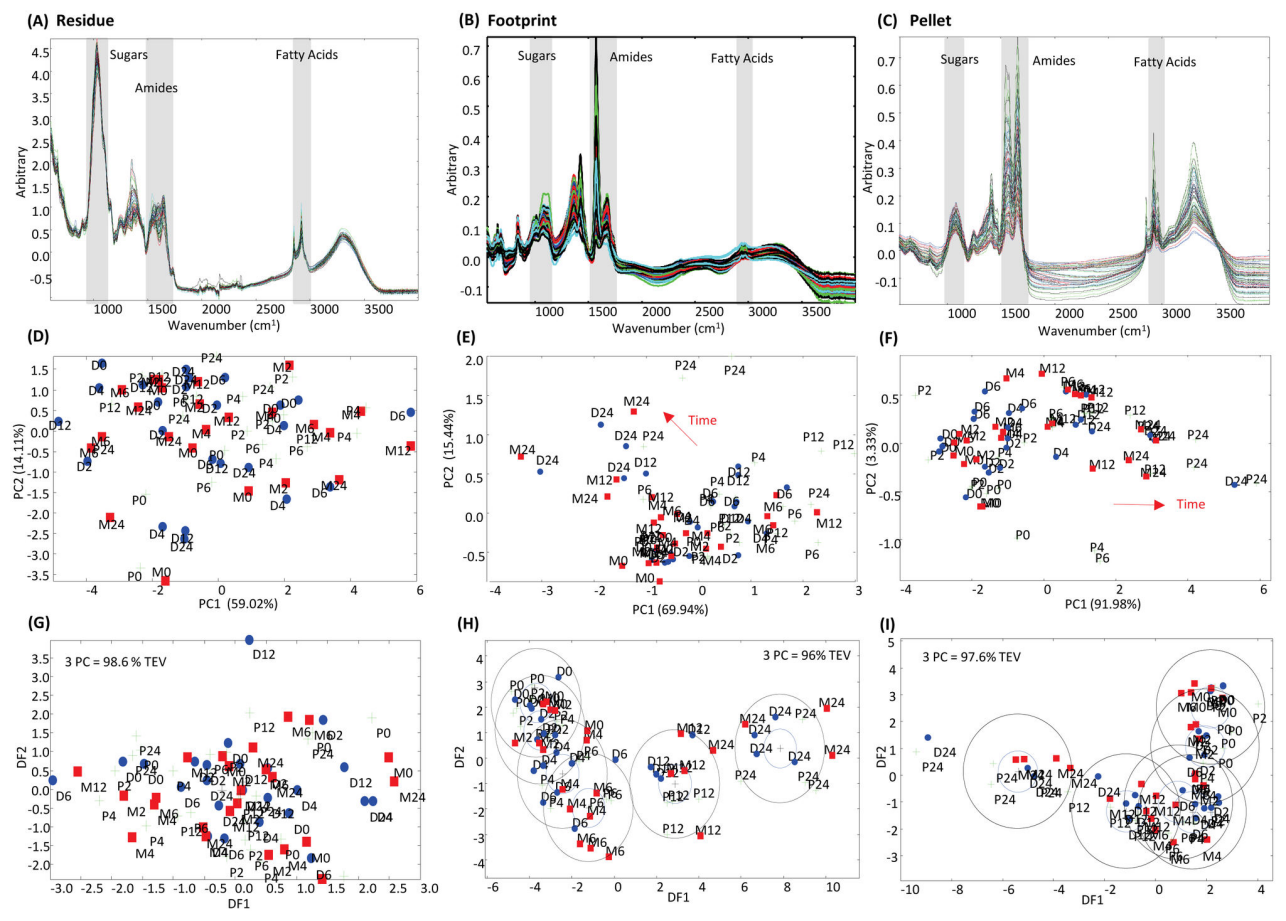
Metabolite fingerprinting of rumen interactome fractions using Fourier Transform Infra-Red spectroscopy. Fourier Transform Infra-Red (FTIR) spectra of fractions of rumen microbe-plant fermentation mixes fractionated into (**A**) residue (plant residue including colonising micro-organisms), (**B**) pellet (bacterial pellet recovered from planktonic phase) and (**C**) footprint (cell free planktonic phase liquor). Areas of the spectra corresponding to discrete chemical groups are highlighted in grey; sugars (wavenumbers 900-950 cm^-1^), amides (wavenumbers 1680-1655 cm^-1^ [Amide I] and 1530-1550cm^-1^ [Amide II]) and fatty acids (wavenumbers 3100-2800 cm^-1^). Principal Component Analyses (PCA) of FTIR spectra from (**D**) residue, (**E**) footprint and (**F**) pellet samples. Discriminant Function Analyses (DFA) based on 3 PCs from FTIR spectra for (**G**) residue, (**H**) footprint and (**I**) pellet samples. Total explained variance (TEV) by 3 PCs for each DFA model is indicated. Green/ P = Premium; blue/ D = AberDart; red/ M = AberMagic. 0, 2, 4, 6 12 and 24 refers to the corresponding hours after addition of rumen microbes to plant genotypes.

In order to get a more complete understanding of the metabolism of the microbe-substrate interactome underlying these changes in fermentation, the fermentation mixes were fractionated into residue (plant residue including colonising micro-organisms), pellet (bacterial pellet recovered from planktonic phase) and footprint (cell free planktonic phase liquor) and analysed by FTIR (Figure 1A-C). Areas of the spectra corresponding to discrete chemical groups [30] are highlighted in grey: the pyranose ring of sugars (wavenumbers 900-950 cm^-1^), amides (wavenumbers 1680-1655 cm^-1^ [Amide I] and 1530-1550cm^-1^ [Amide II]) and fatty acids (wavenumbers 3100-2800 cm^-1^). These indicated the predominance of sugars in the residue (Figure 1A), amides in the footprint (Figure 1B) and pellet (Figure 1C), but also the relative lack of fatty acids in the footprint (Figure 1B). Principal component (PCA) and Discriminant Function Analyses (DFA) were applied to the FTIR spectral data to resolve major areas of similarity and difference occurring during the fermentations of the three grass genotypes (Figure 1D-I). Although PC 1 and 2 cumulatively accounted for 73 % of the variation present in residue samples it was not possible to discriminate between the three grass varieties (Figure 1D). Footprint samples showed an effect of time regardless of grass genotype, with DFA suggesting a slight separation of samples containing Premium from those containing either AberDart or AberMagic (Figure 1E, H). Similarly, time was a major effector of differences in pellet samples (Figure 1F) which could be further separated by DFA indicating clustering of early (up to 6 h) and late (12 and 24 h) samples regardless of grass variety included in the incubations (Figure 1I).

**Figure 2:**
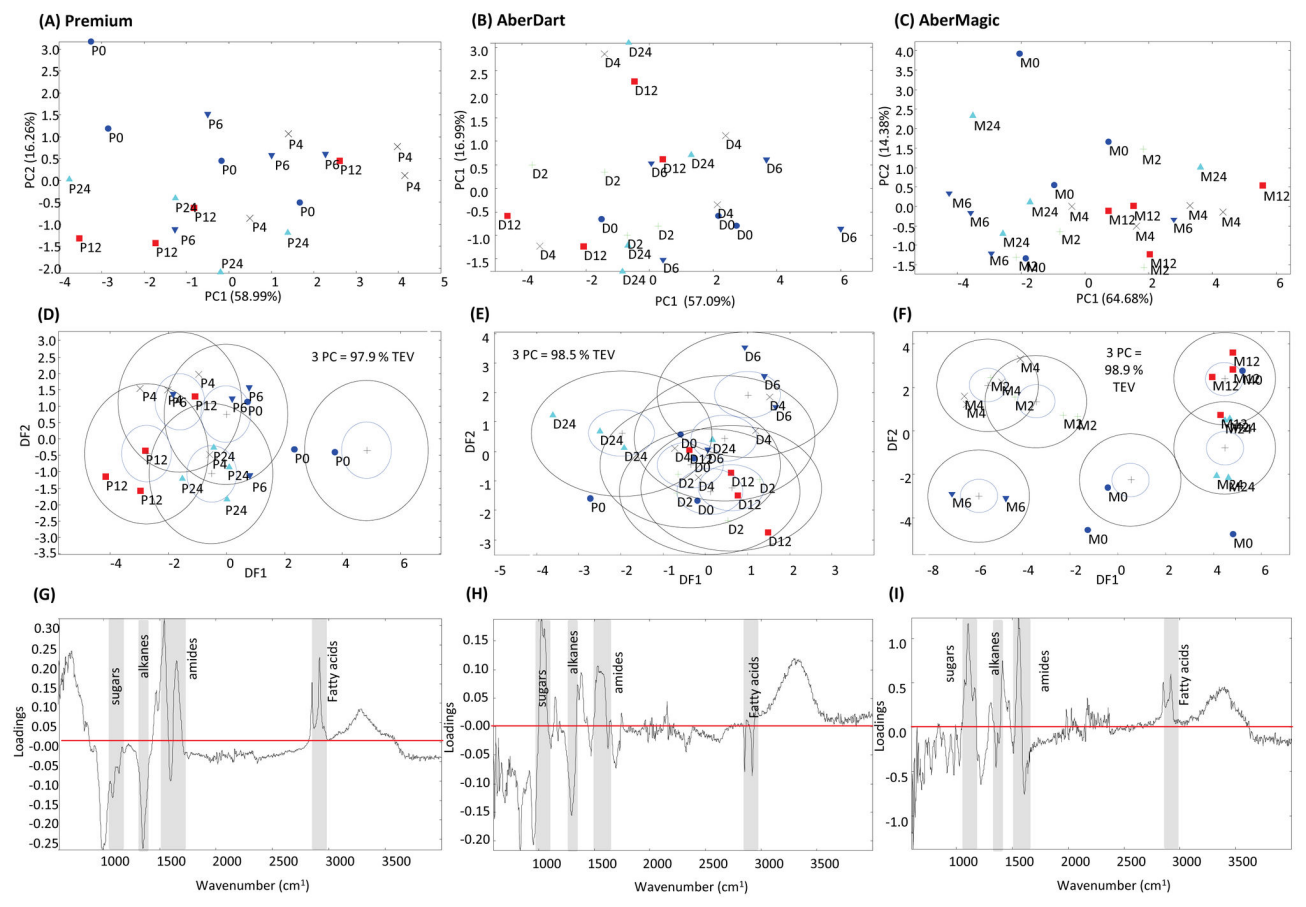
Metabolite fingerprinting of the residue fraction of the rumen microbe-plant genotype interactome. Principal Component Analyses (PCA) of FTIR spectra from (**A**) Premium, (**B**) AberDart and (**C**) AberMagic. Discriminant Function Analyses (DFA) based on 3 PCs for (**D**) Premium, (**E**) AberDart and (**F**) AberMagic. Total explained variance (TEV) by 3 PCs for each DFA model is indicated. P = Premium; D = AberDart; M = AberMagic. 0 (blue circles), 2 (green cross), 4 (black cross), 6 (blue down triangle), 12 (red square) and 24 (cyan triangle) refers to the corresponding hours after addition of rumen microbes to plant genotypes. The loading vectors indicating major sources of variation within DF1 in (**G**) Premium, (**H**) AberDart and (**I**) AberMagic. The horizontal red lines (G, H, I) indicate the points where wavenumbers are making no contribution to DF1. Areas of the spectra corresponding to discrete chemical groups are highlighted in grey; sugars (wavenumbers 900-950 cm^-1^), amides (wavenumbers 1680-1655 cm^-1^ [Amide I] and 1530-1550cm^-1^ [Amide II]), fatty acids (wavenumbers 3100-2800 cm^-1^) and substituted alkanes (wavenumbers 1750-1800 cm^-1^).

 Separate analysis of the FTIR fingerprints for each fraction according to plant variety proved to be particularly revealing. It was clear that the residue samples from three genotypes behaved differently in regard to discrimination between timepoints. In fermentations containing Premium, apart from 0 h samples, no discrimination between timepoints was observed by either PCA or DFA (Figure 2A, D). With AberDart a subtle effect of time could be seen in DFA alone (Figure 2B, E) whilst with AberMagic good separation using DFA was seen along DF1 axis reflecting a differential between early (0-6 h) and late (12-24 h) acting effects (Figure 2C, F). Loadings plots display how strongly a particular variable correlates with a particular Principal Component (PC) or Discriminant Function (DF) i.e. the relative importance of a variable in deriving the projections shown in the PCA or DFA plots. Thus, it was significant that loadings plots showed that sugars were major sources of variation for the three genotypes (Figure 2G-I). The fact that positive and negative loadings plots were returned simply shows their relative correlation with a PC in a particular analysis. As the PCs differ in each PCA their relative signs in comparing between PCAs is of no biological significance. 

**Figure 3:**
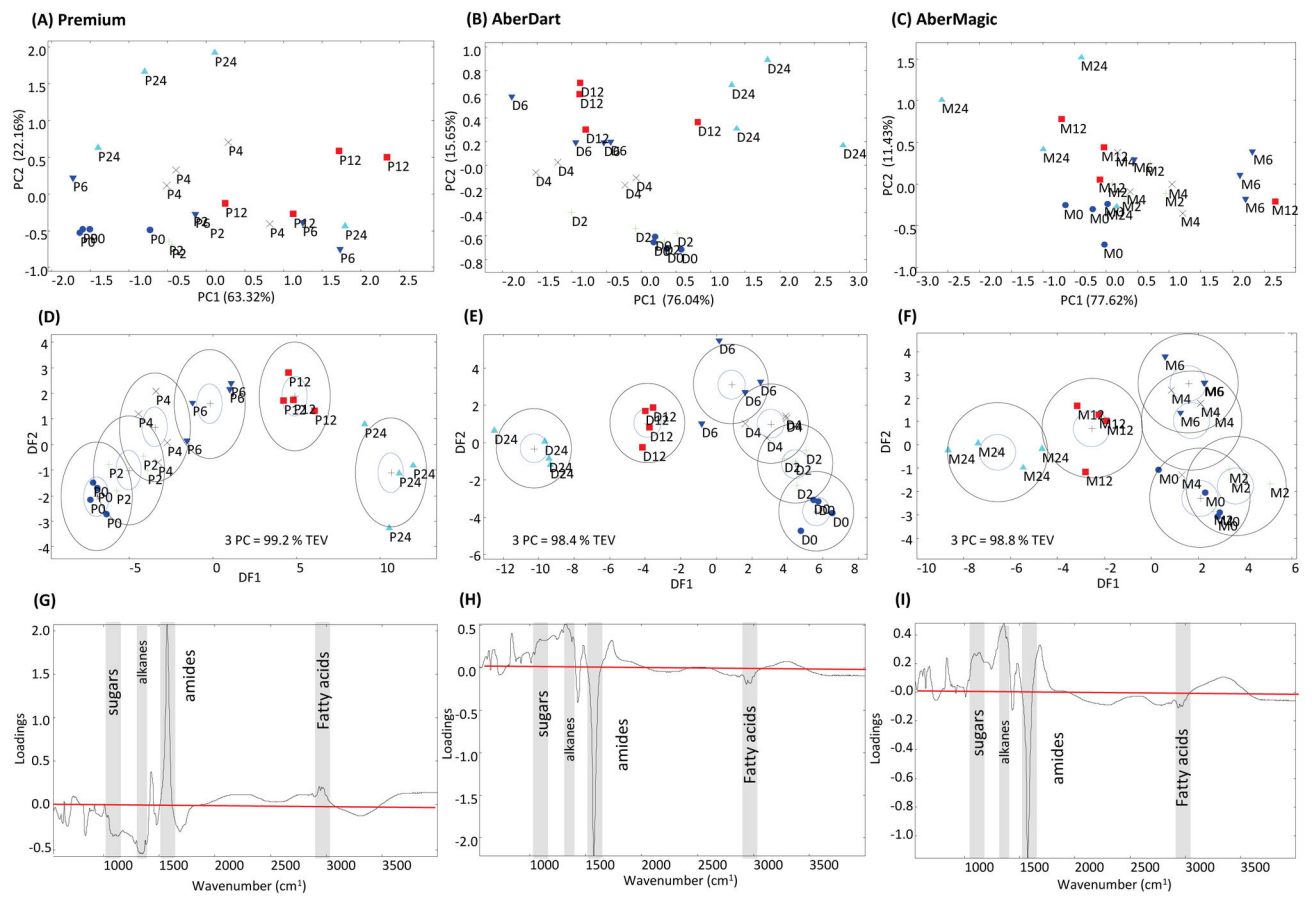
Metabolite fingerprinting of the footprint fraction of the rumen microbe-plant genotype interactome. Principal Component Analyses (PCA) of FTIR spectra from (**A**) Premium, (**B**) AberDart and (**C**) AberMagic. Discriminant Function Analyses (DFA) based on 3 PCs for (**D**) Premium, (**E**) AberDart and (**F**) AberMagic. Total explained variance (TEV) by 3 PCs for each DFA model is indicated. P = Premium; D = AberDart; M = AberMagic. 0 (blue circles), 2 (green cross), 4 (black cross), 6 (blue down triangle), 12 (red square) and 24 (cyan triangle) refers to the corresponding hours after addition of rumen microbes to plant genotypes. The loading vectors indicating major sources of variation within DF1 in (**G**) Premium, (**H**) AberDart and (**I**) AberMagic. The horizontal red lines (G, H, I) indicate the points were wavenumbers are making no contribution to DF1. Areas of the spectra corresponding to discrete chemical groups are highlighted in grey; sugars (wavenumbers 900-950 cm^-1^), amides (wavenumbers 1680-1655 cm^-1^ [Amide I] and 1530-1550cm^-1^ [Amide II]), fatty acids (wavenumbers 3100-2800 cm^-1^) and substituted alkanes (wavenumbers 1750-1800 cm^-1^).

**Figure 4:**
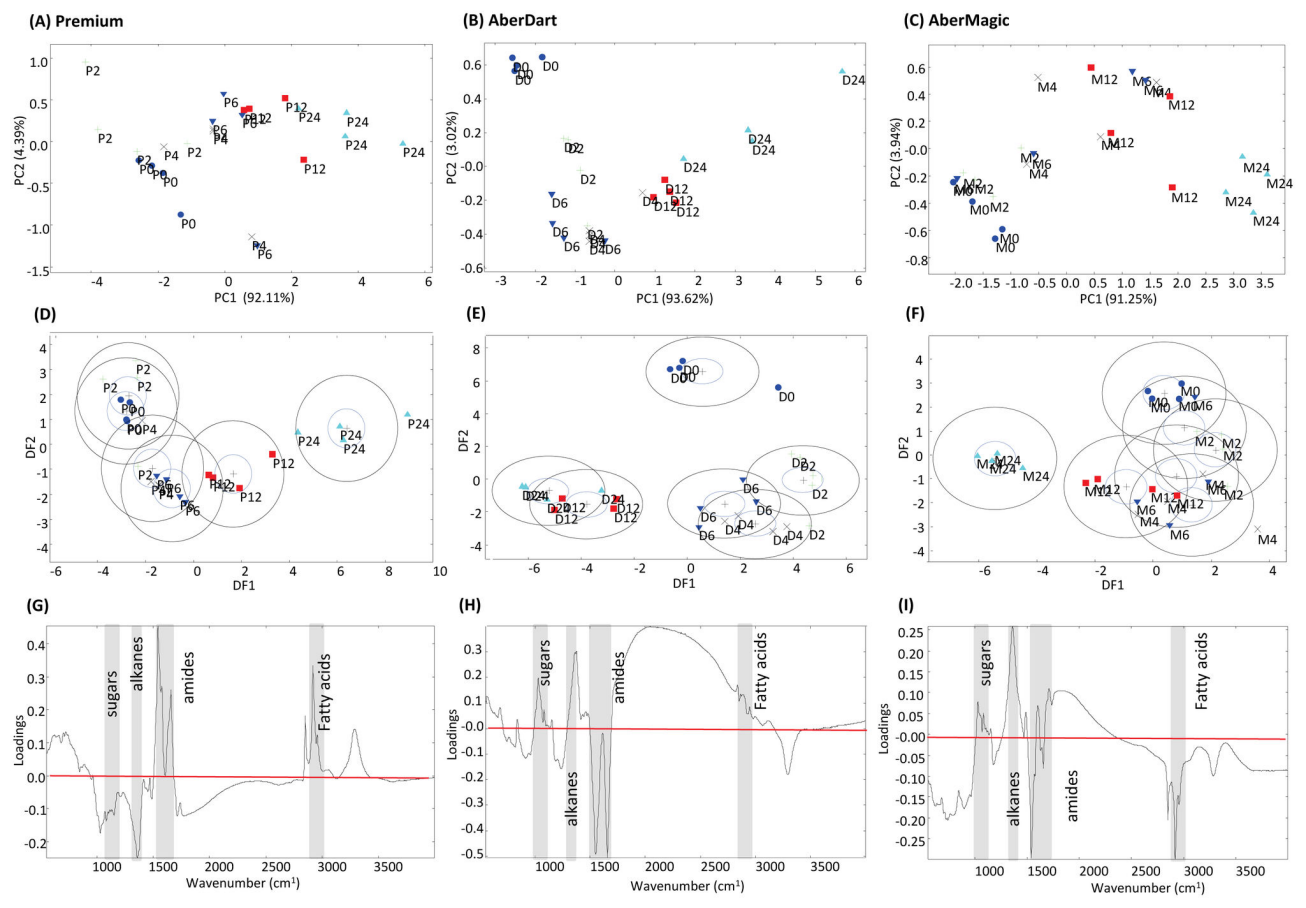
Metabolite fingerprinting of the pellet fraction of the rumen microbe-plant genotype interactome. Principal Component Analyses (PCA) of FTIR spectra from (**A**) Premium, (**B**) Aber Dart and (**C**) AberMagic. Discriminant Function Analyses (DFA) based on 3 PCs for (**D**) Premium, (**E**) AberDart and (**F**) AberMagic. Total explained variance (TEV) by 3 PCs for each DFA model is indicated. P = Premium; D = AberDart; M = AberMagic. 0 (blue circles), 2 (green cross), 4 (black cross), 6 (blue down triangle), 12 (red square) and 24 (cyan triangle) refers to the corresponding hours after addition of rumen microbes to plant genotypes. The loading vectors indicating major sources of variation within DF1 in (**G**) Premium, (**H**) AberDart and (**I**) AberMagic. The horizontal red lines (G, H, I) indicate the points were wavenumbers are making no contribution to DF1. Areas of the spectra corresponding to discrete chemical groups are highlighted in grey; sugars (wavenumbers 900-950 cm^-1^), amides (wavenumbers 1680-1655 cm^-1^ [Amide I] and 1530-1550cm^-1^ [Amide II]), fatty acids (wavenumber 3100-2800 cm^-1^) and substituted alkanes (wavenumbers 1750-1800 cm^-1^).

In contrast to the residues, multivariate analyses of footprint samples (the cell-free planktonic liquor) clearly showed a discrimination of samples by incubation time with a progression from 0 to 24 h samples across the axes (Figure 3A-F), albeit left to right in Premium but right to left in AberDart and AberMagic across DF1. In addition, there was separation across DF2 from top to bottom, particularly for AberDart. The changes in the metabolome of the footprint over time are assumed to represent the incorporation of substrate into microbial growth. Discrimination of pellet samples according to time was obvious with all genotypes although the timing of separation between changes occurring early and later in fermentation was genotype specific (Figure 4). Time-dependent clustering was particularly obvious with samples where AberDart was provided as the substrate; three groupings (at 0 h, at 2, 4 and 6 h and at 12 and 24 h) were revealed by DFA (Figure 4E). As seen with footprint samples, the components with the most variable region (determined by loading score, Figure 4G-I) of the FTIR spectra were sugars, alkanes, amides and fatty acids. (Figure 4G-I).

**Figure 5:**
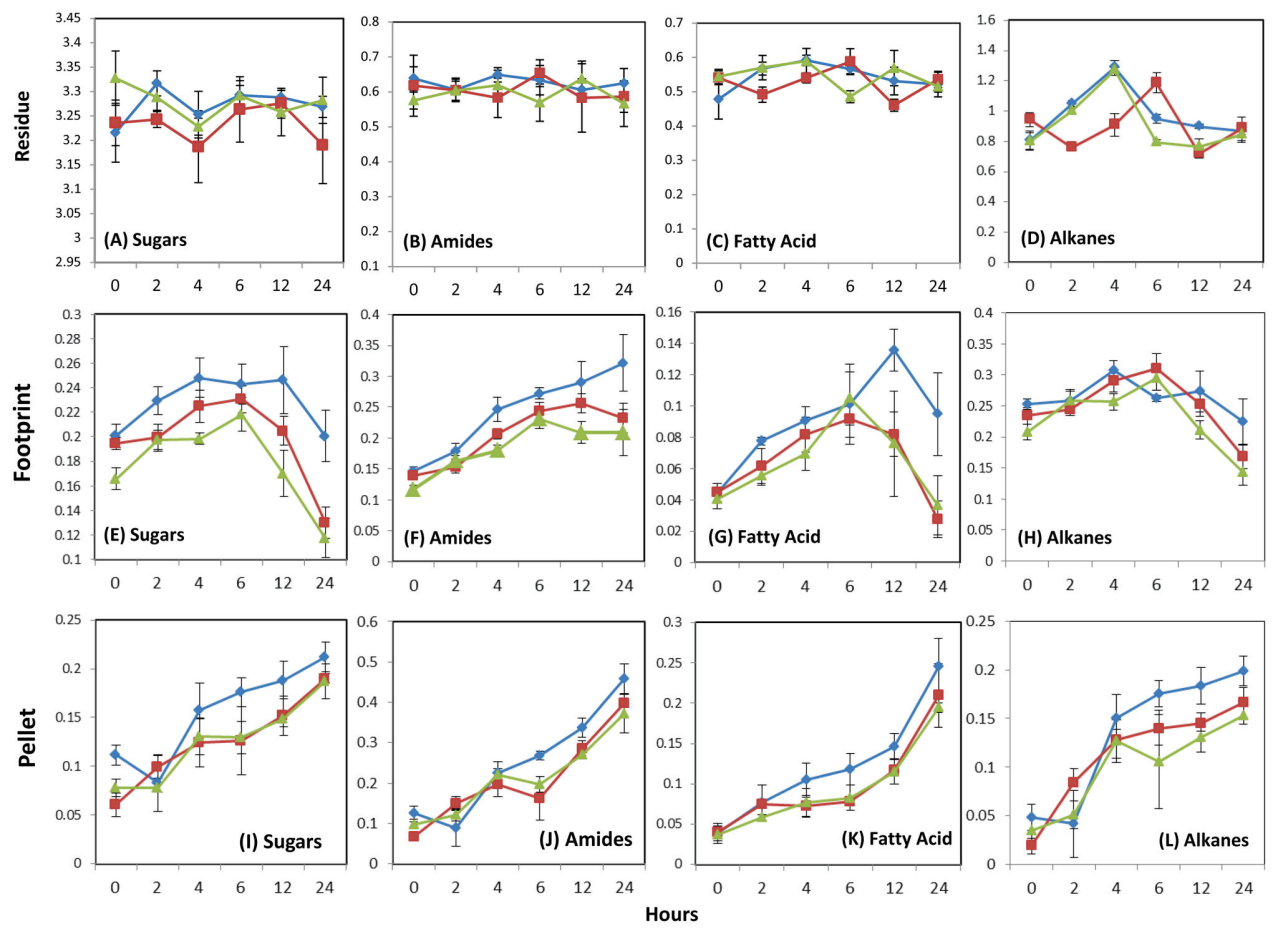
Changes in metabolite groups in rumen interactome fractions as revealed using Fourier Transform Infra-Red spectroscopy. Absorbancies of areas of the spectra corresponding to discrete chemical groups are highlighted in grey; sugars (wavenumbers 900-950 cm^-1^), amides (wavenumbers 1680-1655 cm^-1^ [Amide I] and 1530-1550cm^-1^ [Amide II]), fatty acids (wavenumbers 3100-2800 cm^-1^) and substituted alkanes (wavenumbers 1750-1800 cm^-1^) were extracted for spectra of residue, pellet and footprint fractions of rumen microbe-plant fermentation mixes “residue” (plant residue including colonising micro-organisms). Mean values +/- SE (n = 6) are plotted for each interactome based on input of plant genotypes: Premium (blue diamond), AberMagic (green triangle) and AberDart (red square).

Given that the majority of the variation between spectra for residue (Figure 2G-I), footprint (Figure 3G-I) and pellet (Figure 4G-I) was present in the regions of FTIR spectra relating to sugars, alkanes, amides and fatty acids, the mean absorbancies for these regions were calculated. This enabled a more detailed, statistical investigation of the changes in composition in the different fractions of the interactome during fermentation. In the residue fractions, no statistically significant (data not shown) differences were observed with the exception of alkanes (Figure 5A-D). The alkane content of the residues increased over the first 4 h in incubations where Premium and AberMagic were provided as substrates, with this increase happening between 2 and 6 h in AberDart (Figure 5D). In contrast, footprint profiles were significantly different by substrate genotype for all parameters apart from alkanes. Sugar content of the footprint signal was highest in Premium and lowest in AberMagic throughout the incubation period (Figure 5E). ANOVA revealed significant differences between means according to genotype and time (both P <0.001) and there was a genotype x time interaction (P <0.05). In all cases the sugar signal increased until 6 h, after which it decreased; most rapidly where AberMagic was supplied as substrate but only slightly when Premium was the substrate. Amide signal in the footprints was also significantly different according to genotype and time (both P <0.001) and there was a genotype x time interaction (P <0.05). Amide accumulated progressively in all genotypes but was greatest in Premium and lowest in AberMagic (Figure 5F). The mean fatty acid levels of the footprint samples was similar until 6 h after which it decreased in incubations containing AberMagic and AberDart, but continued to increase further until 12 h where Premium was the substrate (Figure 5G). In consequence ANOVA revealed significant differences by genotype and time but no significant interaction. No significant differences were detected in the alkane contents of the footprint samples. In the pellet samples (Figure 5I, J, K, L) increases in signal from the four parameters (sugars, amides, fatty acids and alkanes) increased progressively with increasing incubation time regardless of genotype, reflecting increased planktonic microbial growth. ANOVA revealed genotype dependent significant differences in sugars and both genotype and time dependent significant differences in amides, fatty acids and alkanes (all P <0.005). Interactions between effects of time and genotype were detected for amides and alkanes (P = 0.05). In general, signals arising from incubations containing Premium as the substrate exceeded those from incubations with either AberMagic or AberDart and this was most obvious after 4 h incubation.

## Discussion

The genotype of forage fed to ruminants is known to affect outputs, presumably by alteration at the level of the rumen microbiota [1,2,31,32]. This is especially relevant in the context of grazing where post-ingestion plant responses to the rumen environment can potentially alter substrate composition and limit microbial growth [20,33,34]. One of the main features of the rumen microbial ecosystem is its complexity, but a limited number of pure culture isolates in relation to the number of 16S rRNA sequences that can be detected in rumen samples [6,35] restricts our current ability to apply predictive biology to strategies for improvement of feed for ruminants and progress towards more sustainable production systems. The aim of this work was to explore interactomic approaches to assist in the development of novel, forage based approaches to improved ruminant nutrition. Our approach was based on FTIR as an inexpensive, non-discriminatory technique. Previous metabolomic investigations of the rumen have all studied the steady state, via the cell-free metabolome (for example 36) which is equivalent to our footprint fraction. Our FTIR-fingerprint based approach involves no sample extraction or fractionation steps and so provides non-targetted and unbiased metabolite fingerprints of the different factions of the whole rumen interactome.

Measurements of fermentation end products (ammonia and VFA) can inform about suitability of substrates for fermentation and have been used effectively in screening substrates. However, these measurements do not give any information about what components of the (chemically complex) substrate are responsible for the underlying biology of any differences observed. It is important to understand the drivers of fermentation if we are to achieve feed-based improvements in production efficiency. This is where non-discriminatory, hypothesis-forming approaches such as FTIR can be of use in establishing the compounds and biological mechanisms underlying resultant changes in fermentation outputs more effectively than traditional, hypothesis-based targetted approaches.

 The entire FTIR signal was rich and complex (Figure 1) and an underlying structure to the data was indicated that required further statistical analysis. Thus, loadings were used to identify sources of maximum variation between the spectra. While not providing data for individual metabolites, broad chemical groupings could be defined by FTIR fingerprinting which when used in a dual metabolomics approach were used as a surrogate for flux to explore the relationships between delivery of nutrients by the forage and its utilisation by the rumen microbiota. The power of the FTIR technique is shown by the observation that the same microbial inoculum showed differences in patterns of utilisation of substrate carbohydrate and protein from grass varieties that would not usually be considered significantly different according to results of proximate analysis (Figure 5, Table 1). This was most pronounced for the residue samples where, broadly, DF1 showed genotype specific differences in timings relating to separation of the early and late events in colonisation and fermentation (Figure 2). According to FTIR, during the first 6 h of fermentation the flux of key nutrients between forage and bacteria depended on source genotype in a manner that would not have been predicted from ammonia and VFA measurements alone; no effects of genotype were detected for total or individual VFA (Table 2) and genotype dependent differences in total VFA and ammonia were only detectable at 24 h (Table 3). This suggests that events occurring early in the development of the fermentative interactome can be defined by forage genotype and can have implications for fermentation outputs over the longer term. For example ammonia accumulation in the liquid samples was greatest in AberMagic which would suggest the greatest microbial protein synthesis. However, this did not correspond with the increase in amide signal in the pellet which was highest in Premium. This suggests that availability of non-ammonia amide forms (eg. peptides) are driving microbial N incorporation.

Grass genotype specific differences in development of the interactome were reflected in time dependent separation in residue, footprint and pellet samples (Figures 2-4), reflecting flux of metabolites from residue into microbial growth in the planktonic phase of the ecosystem. The residue sample contained both the plant residue and the attached bacterial community and so this fraction can be considered as a sub-interactome within the entire ecosystem and this was reflected in the original data-rich spectra generated from these samples (Figure 1). Attachment of rumen microbiota to ingested forage is central for utilization of plant nutrients and numerous studies have shown that ruminal bacteria colonise fresh forage quickly [37–40]. In the work by Allwood et al. [23], bacteria were effectively removed from the cell culture samples by sequential washing in 0.2 % NaCl. Although there would be merit in separation of plant and attached bacteria in future studies, separation of colonising rumen bacteria from the forage substrate is difficult, usually requiring some form of chemical and physical treatment to enable detachment of a representative bacterial pool [41–43]. In the current study it was considered that such processing could substantially alter the plant metabolome and so provide an erroneous impression of the interactome under investigation. Therefore, analysis of the residue represents the net effect of multiple interactions occurring between micro-organisms which are colonising a forage substrate which is changing in composition in response to the rumen environment [20,21,37,38,41]. Analysis of the loading scores indicated that sugars and fatty acids were the major sources of variation in discriminant function (DF) between times and genotypes. Together, these represent both that major changes in metabolism occurred within this interactome during the course of the fermentation, and also that the interface formed between the plant cells and colonising bacteria was substantially different during development of the fermentations of the three grass genotypes. As all incubations were inoculated with the same mixed inoculum, the observed differences were presumably due to different nutrient availabilities which would provide different niches for the colonising microbiota and could affect successional ecology in the rumen ecosystem [37,41]. Forage-driven differences in ruminal metabolism have been reported previously. *In vitro* fermentation of red clover and ryegrass led to different microbial populations, with more bacteria, fungi and methanogens produced in fermentations involving ryegrass [44]. It is proposed therefore, that the effect of forage composition (or post-ingestion responses of plant cells to the rumen) on the metabolic activities of colonising micro-organisms will also have consequences in terms of nutrient supply and function of the planktonic phase micro-organisms.

While signal intensity for all spectral regions increased progressively in the pellet samples, there was an approximate tenfold difference between sugar signals arising from residue and those present in footprint or pellet (Figure 5A, E, I) with available sugars eventually depleted from the medium. The correspondence of greater VFA in incubations in which AberMagic was supplied as the substrate when compared with AberDart or Premium as substrates is consistent with the enhanced depletion of sugars from the footprint at all incubation times (Figure 5E) and suggests that the genetically determined composition of the forage substrate AberMagic promoted rapid fermentation, and by implication enhanced microbial growth. However, it is clear that either relatively little of the available total carbohydrate from the plant substrate was released to the footprint for microbial uptake, or carbohydrate was only transiently present in the footprint, being immediately taken up by the planktonic bacteria. The latter seems likely from the linear (rather than exponential) increase in the carbohydrate signal originating from the bacterial pellet and previous results [33]. Also, as availability of soluble carbohydrate affects plant cell survival under anoxic and hypoxic conditions [45–48] so reserves of cellular carbohydrate in plant cells could be quickly depleted on exposure to the rumen environment (anaerobic, 39 °C) due to demands from *de novo* synthesis of stress-related proteins. This suggests that attention to availability as well as content of plant carbohydrates should be an important consideration in designing forage breeding targets. The asynchrony hypothesis [49] suggests that when feeding is initiated ammonia builds up in the rumen when carbohydrate is limiting so theoretically increased efficiency of protein utilisation can be achieved by increasing the soluble carbohydrate content of the feed [32,49]. Here there was no evidence of asynchrony in the initial post-incubation period but rather, there was a co-accumulation of sugars and amides in both the cell free medium (footprint sample) and planktonic bacteria (pellet sample), despite a relatively low forage carbohydrate composition (Table 1) which would be typical of that found in spring growth [50]. However, the sugar signal of the FTIR fingerprint does not discriminate between structural and non-structural (soluble) carbohydrate so reflects hemi/cellulose and bacterially-derived glycans as much as simple sugars (hexoses, sucrose). 

## Conclusions

In conclusion, analysis of FTIR fingerprinting indicated that rumen micro-organisms showed substrate-related metabolic differences within the plant-rumen interactome during fermentations when the substrates were compositionally similar, but genetically distinct fresh forages. Differences in the plant-attached bacterial interactome translated through to supply of nutrients from plant cells to the medium to drive microbial growth in the planktonic phase. These subtle interactions of apparently similar forage materials could contribute to observed mismatches in apparent nutrient supply and predicted rumen function during animal based feed trials. This work with FTIR has therefore established that full metabolite profiling is a logical next step to probe the individual components of these broad chemical groups in more detail. Comparisons of different plant substrates at species or genus level would no doubt reveal larger differentials in substrate utilisation and the potential for novel parameters for consideration. 

## Supporting Information

Figure S1 (TIF)
