# Supplementary figures and images for: Comparative Metabolite Fingerprinting of the Rumen System during Colonisation of Three Forage Grass (Lolium perenne L.) Varieties
Source: PLoS One. 2013 Nov 27;8(11):e82801. doi: 10.1371/journal.pone.0082801 (PMC3842282; doi:10.1371/journal.pone.0082801)

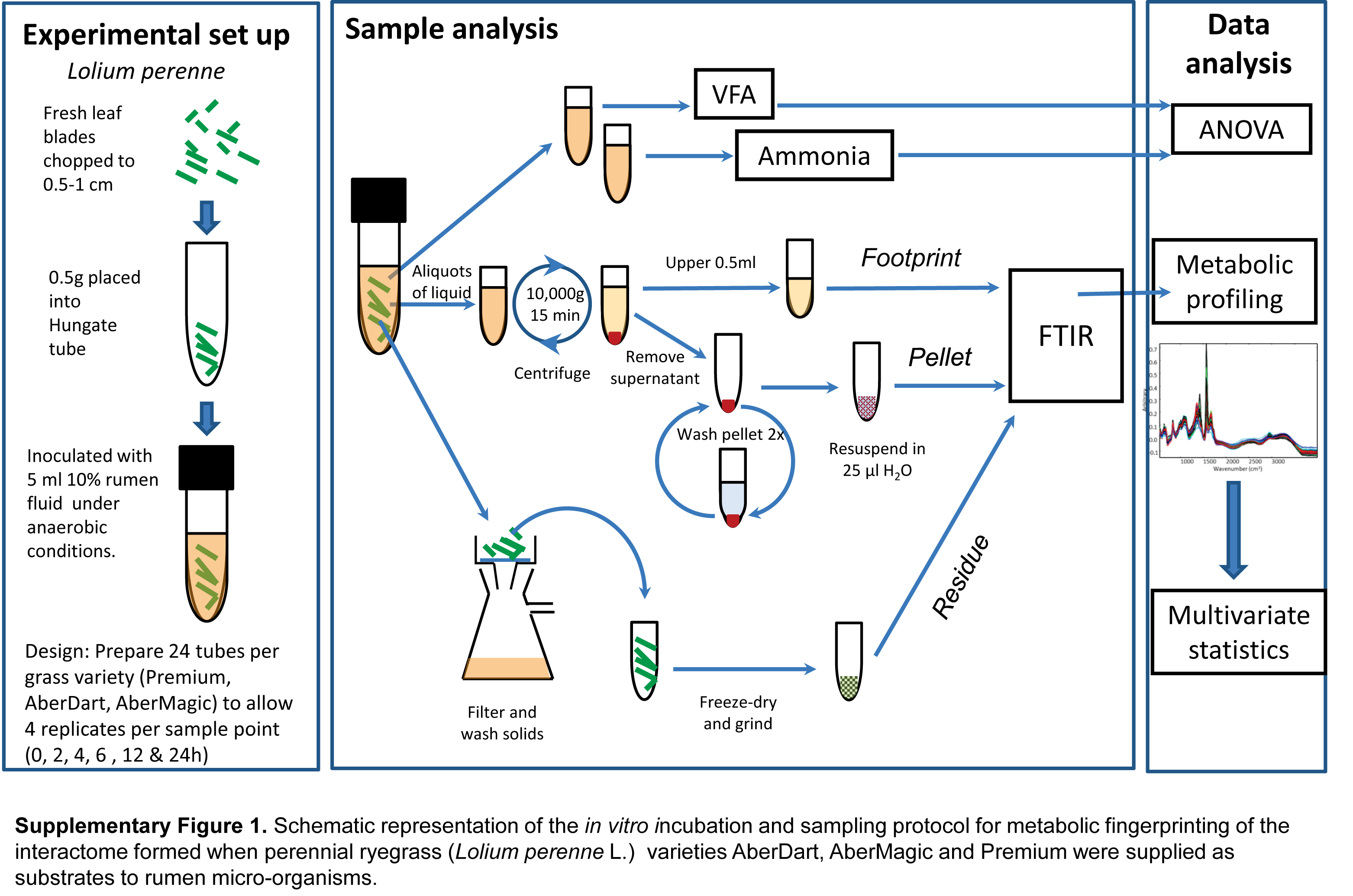

Supplement: Figure S1 — (TIF) [file pone.0082801.s001.tif]
